# In Vivo Measurement of Neurochemical Abnormalities in the Hippocampus in a Rat Model of Cuprizone-Induced Demyelination

**DOI:** 10.3390/diagnostics11010045

**Published:** 2020-12-30

**Authors:** Do-Wan Lee, Jae-Im Kwon, Chul-Woong Woo, Hwon Heo, Kyung Won Kim, Dong-Cheol Woo, Jeong Kon Kim, Dong-Hoon Lee

**Affiliations:** 1 Department of Radiology, Asan Medical Center, University of Ulsan College of Medicine, Seoul 05505, Korea; medimash@gmail.com (K.W.K.); kim.jeongkon@gmail.com (J.K.K.); 2 Convergence Medicine Research Center, Asan Institute for Life Sciences, Asan Medical Center, Seoul 05505, Korea; kji1733@naver.com (J.-I.K.); wandj79@hanmail.net (C.-W.W.); dcwoo@amc.seoul.kr (D.-C.W.); 3 Department of Convergence Medicine, Asan Medical Center, University of Ulsan College of Medicine, Seoul 05505, Korea; heohwon@gmail.com; 4 Department of Radiation Convergence Engineering, Yonsei University, Wonju 26493, Korea

**Keywords:** rat brain, demyelination, hippocampus, metabolites, multiple sclerosis

## Abstract

This study quantitatively measured the changes in metabolites in the hippocampal lesions of a rat model of cuprizone-induced demyelination as detected using in vivo 7 T proton magnetic resonance spectroscopy. Nineteen Sprague Dawley rats were randomly divided into two groups and fed a normal chow diet or cuprizone (0.2%, w/w) for 7 weeks. Demyelinated hippocampal lesions were quantitatively measured using a 7 T magnetic resonance imaging scanner. All proton spectra were quantified for metabolite concentrations and relative ratios. Compared to those in the controls, the cuprizone-induced rats had significantly higher concentrations of glutamate (*p* = 0.001), gamma-aminobutyric acid (*p* = 0.019), and glutamate + glutamine (*p* = 0.001); however, creatine + phosphocreatine (*p* = 0.006) and myo-inositol (*p* = 0.001) concentrations were lower. In addition, we found that the glutamine and glutamate complex/total creatine (*p* < 0.001), glutamate/total creatine (*p* < 0.001), and GABA/total creatine (*p* = 0.002) ratios were significantly higher in cuprizone-treated rats than in control rats. Our results showed that cuprizone-induced neuronal demyelination may influence the severe abnormal metabolism in hippocampal lesions, and these responses could be caused by microglial activation, mitochondrial dysfunction, and astrocytic necrosis.

## 1. Introduction

Multiple sclerosis (MS) is a chronic and progressive inflammatory disease of the central nervous system (CNS) [1,2]. The most significant characteristic features of MS are inflammatory lesions, primary demyelination, axonal damage, plaques, microglial activation, mitochondrial dysfunction, and abnormal metabolism [2,3,4]. In recent years, studies have shown that demyelination affects not only the white matter (WM) in the corpus callosum but also the gray matter (GM), such as that in the cortex, hippocampus, and cerebellum [5,6]. Among these brain regions, the hippocampus plays a pivotal role in learning processes, spatial memory, and the consolidation of long-term memory from short-term memory [7,8]. The hippocampus was chosen as the major target of the present study, since MS patients commonly present with cognitive deficits, often in the form of episodic memory impairments, which might be caused by pathophysiological changes (functional/metabolic abnormalities) in this region [2,9].

To identify key markers of the complex mechanisms in MS, various animal models, including toxin-induced models, have been established [10,11]. In particular, cuprizone is a selective and sensitive copper chelating agent that is commonly used to create a toxicity-induced non-autoimmune animal model of MS to assess the pathophysiological processes of cerebral demyelination and remyelination [3]. Cuprizone induces mature oligodendrocyte damage with subsequent demyelination, but it does not damage other cell types in the CNS [10,11]. Moreover, previous studies have suggested that the cuprizone-induced model is reproducible and sustainable for the neurobiological assessment of cerebral demyelination in MS [3,12]. In this study, we used a well-characterized cuprizone-induced MS model to investigate the neurochemical changes in the hippocampal region.

In vivo magnetic resonance (MR)-based imaging techniques provide a non-invasive method for evaluating various anatomical lesions and structural changes, including focal demyelination, axonal injuries, and inflammation in MS [13]. However, MR-based imaging cannot distinguish between cerebral demyelination, inflammation, and axonal injuries [13,14]. Thus, to obtain a better insight into the underlying pathology and evaluate biological signals from the neurochemical compounds involved in MS, there is a need to reflect molecular processes using in vivo MR-based techniques.

In vivo proton MR spectroscopy (^1^H MRS) is a unique method for non-invasive quantification of cerebral neurochemical markers involved in neuromolecular processes [15,16,17]. High-resolution in vivo ^1^H MRS at 7 T allows the assessment of more detailed chemical compounds with higher precision than lower magnetic field strength [18,19]. In the in vivo MR spectra, the resonance intensities and total area of metabolites are proportionally represented by the cerebral metabolite concentrations. Furthermore, both the absolute concentration of metabolites and their ratios have been used in numerous human and animal studies [20]. The utility of in vivo ^1^H MRS has been previously described [20,21].

The aim of this study was to quantitatively examine neurochemical alterations in hippocampal lesions in a rat model of cuprizone-induced demyelination, detected using ^1^H MRS at 7 T. For this purpose, the present study quantitatively assessed changes in the concentrations and metabolic signal ratios of detectable neurochemical profiles in the hippocampal region of the rat brain.

## 2. Materials and Methods

### 2.1. Animals and Cuprizone Intoxication

All animal experiments were approved by the Institutional Animal Care and Use Committee at the Asan Medical Center, University of Ulsan College of Medicine (No. 2018-13-271). The animal experiments were carried out according to the “Guide for the Care and Use of Laboratory Animals” (NIH Publications No. 80-23, revised 1978).

Nineteen Sprague Dawley rats were purchased (Orient Bio Inc., Seongnam, Kyunggi-do, Korea) and divided into two groups (control (CTRL) group; n = 11, and cuprizone-induced (CPR) group; n = 8). The animals were housed in standard plastic cages and maintained on a 12-h light-dark cycle at an ambient temperature of 23–24 °C. Before the start of the experiments, the animals were allowed free access to food and water for a week. To induce demyelination in the hippocampus, eight CPR rats were fed a milled diet with 0.2% cuprizone (bis[cyclohexanone]oxaldihydrazone, #370-81-0, Merck KGaA, Darmstadt, Germany) for 7 weeks, while CTRL rats were maintained on a regular chow diet.

### 2.2. In Vivo ^1^H MR Spectroscopy

**Figure 1:**
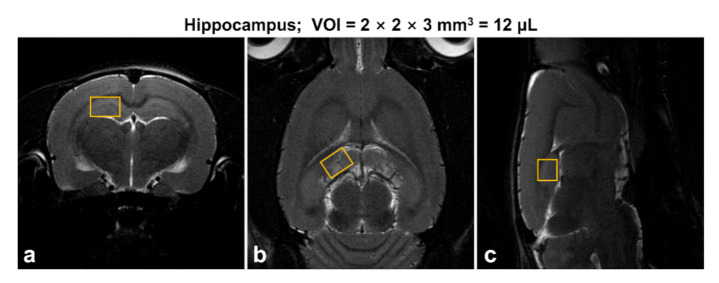
Representative multi-slice T2-weighted magnetic resonance (MR) images: (**a**) axial, (**b**) coronal, (**c**) sagittal, and voxel placement in the hippocampal region (yellow).

The designs of in vivo ^1^H MRS studies have been previously described [22]. All in vivo MR experiments were performed using a horizontal 7 T/160-mm preclinical MRI scanner (Bruker PharmaScan 70/16, Bruker BioSpin, Ettlingen, Germany) that resonated at 300.32 MHz for ^1^H, equipped with a 400 mT/m self-shielded gradient system. All data were recorded using a receive-only rat brain surface coil in combination with a 72-mm volume coil for excitation. All animals were anesthetized using a nose cone via spontaneous inhalation of 1–2% isoflurane in 70% N_2_O/30% O_2_ (flow rate: 1.0 L/min). For the placement of ^1^H MRS voxel, two-dimensional T_2_-weighted MR images were acquired as follows: repetition time (TR) = 5250 ms, echo time (TE) = 66 ms, field of view = 25 × 25 mm^2^, image matrix = 256 × 256, echo spacing = 11 ms, average = 2, and total scan time = 2 min 48 s per image plane (axial, sagittal, and coronal). Water-suppressed in vivo proton spectra were acquired from the hippocampal region of the right hemisphere (Figure 1). All parameters were as follows: spin-echo-based point-resolved spectroscopy (PRESS) pulse sequence, variable power and optimized relaxation delays method, volume of interest (VOI) = 2 × 2 × 3 mm^3^ (12.0 μL), TR = 5000 ms, TE = 16.3 ms, spectral width = 5000 Hz, average = 256, number of data points = 2048, and total scan time = 23 min 15 s.

### 2.3. Spectral Quantification

Postprocessing of ^1^H MRS raw data was quantified using a linear combination of models (LCModel, v. 6.3-1D, copyright: Stephen W. Provencher, Stephen Provencher Inc., Oakville, Canada) in a fully automated pipeline. In vivo proton spectra were analyzed using a set of simulated basis sets including 18 metabolites, as follows: Ala, alanine; Asp, aspartate; Cr, creatine; GABA, gamma-aminobutyric acid; Glc, glucose; Glu, glutamate; Gln, glutamine; GSH, glutathione; GPC, glycerophosphocholine; Gly, glycine; Lac, lactate; NAA, N-acetylaspartate; NAAG, N-acetylaspartylglutamate; mIns, myo-inositol; PCh, phosphocholine; PCr, phosphocreatine; sIns, scyllo-inositol; Tau, taurine; tNAA, total NAA = NAA + NAAG; Glx, glutamine and glutamate complex = Glu + Gln; tCr, total Cr = Cr + PCr; and tCho, total Cho = GPC + PCh. Eddy current correction in all proton spectra was applied and fitted in the chemical shift range from 4.0 to 0.3 ppm. The unsuppressed water signal was used as an internal reference for water scaling, and metabolite concentrations were acquired (μmol/g). Additionally, the relative metabolite levels of Glx, Glu, and GABA were calculated by dividing the tCr signals and expressed as Glx/tCr, Glu/tCr, and GABA/tCr ratios.

An estimate of uncertainty (Cramer–Rao lower bounds (CRLBs)) with frequency domain fitting was provided by the LCModel and expressed as percent standard deviation (%SD). Individual metabolite signals were used to determine the reliability of the fitting; less than 10% SD was considered acceptable for Glu, mIns, Tau, tCho, tNAA, tCr, and Glx; less than 20% SD was acceptable for Cr, PCr, GABA, Gln, and GSH.

### 2.4. Statistical Analysis

All statistical analyses were performed using PASW Statistics 21 (SPSS Inc., IBM Company, Chicago, IL, USA). The metabolite quantification values (absolute concentrations and peak ratio values), spectral quality measurements (signal-to-noise ratio (SNR), and full-width at half-maximum (FWHM)) were normally distributed for all data (Kolmogorov–Smirnov test of normality, all *p* > 0.05), and independent two-sample *t*-tests were used. Statistical differences were assumed to be significant for *p*-values < 0.05.

## 3. Results

**Figure 2:**
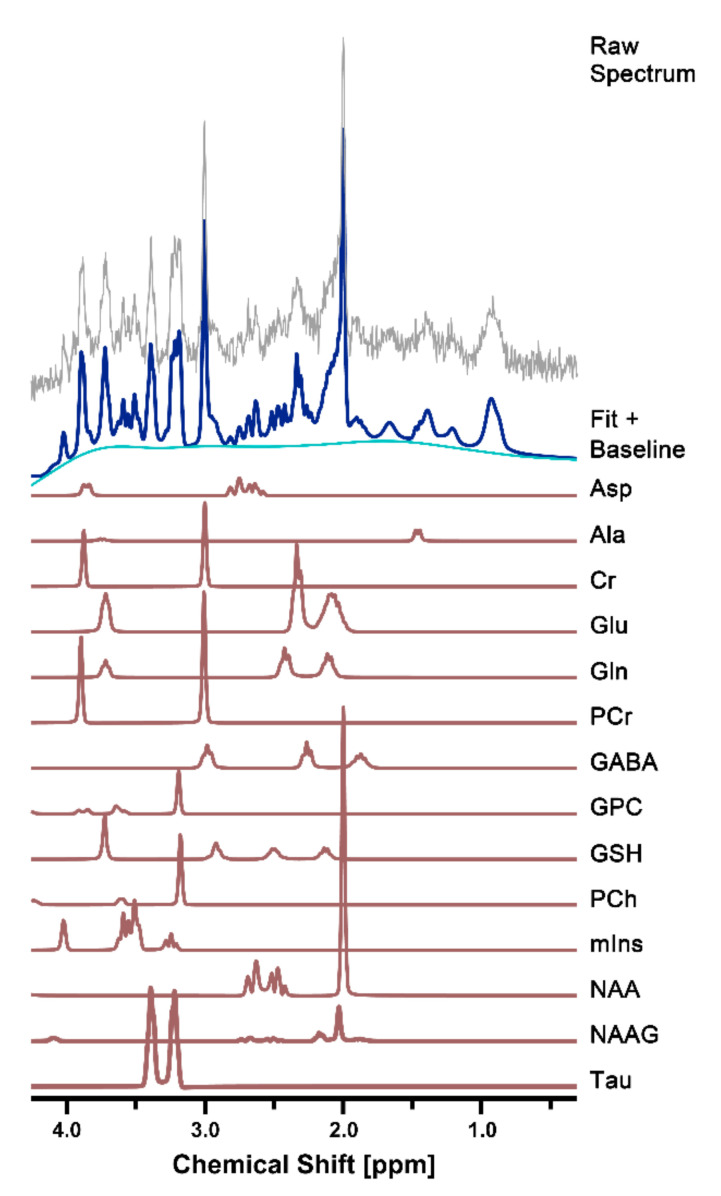
Representative in vivo ^1^H MR spectra in the right hippocampus of cuprizone-treated rats. The figure shows the raw spectrum (gray), fitted spectrum (navy), baseline (light blue), and 14 individual metabolite fits below (brown). Ala, alanine; Asp, aspartate; Cr, creatine; GABA, gamma-aminobutyric acid; Gln, glutamine; Glu, glutamate; GPC, glycerophosphocholine; GSH, glutathione; mIns, myo-inositol; NAA, N-acetylaspartate; NAAG, N-acetylaspartylglutamate; PCh, phosphocholine; PCr, phosphocreatine; ppm, part per million; Tau, taurine.

Representative high-resolution 7 T spectra with narrow linewidths from a single voxel of the hippocampal region were obtained throughout the study. In all proton spectra (Figure 2), the average FWHM (Hz) and SNR values estimated using the LCModel in the CTRL and CPR groups were 7.8 ± 1.0 and 7.5 ± 0.8 (*p* = 0.552) and 13.5 ± 1.0 and 14.0 ± 1.2 (*p* = 0.388), respectively. All spectral quality values showed no significant differences between the two groups.

**Figure 3:**
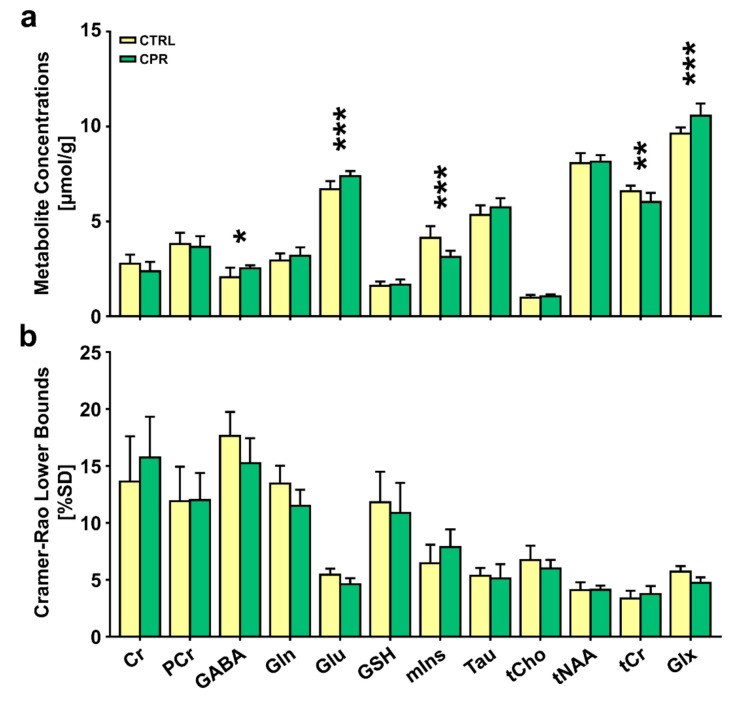
Bar graph indicating the mean cerebral metabolite concentrations (**a**) and Cramer-Rao lower bounds (CRLBs) (**b**) in the right hippocampal region of control (CTRL) and cuprizone-treated (CPR) rats, quantified using Linear Combination of Models software. The vertical lines on each of the bars indicate the (+) standard deviation of the mean values. * *p* < 0.05; ** *p* < 0.01; *** *p* < 0.005. Cr, creatine; GABA, gamma-aminobutyric acid; Gln, glutamine; Glu, glutamate; Glx, Glu + Gln; GSH, glutathione; mIns, myo-inositol; PCr, phosphocreatine; SD, standard deviation; Tau, taurine; tCho (total Cho), GPC (glycerophosphocholine) + PCh (phosphocholine); tCr (total Cr), Cr + PCr; tNAA (total NAA), NAA (N-acetylaspartate) + NAAG (N-acetylaspartylglutamate).

Figure 3 illustrates the assessed cerebral metabolite concentrations and spectral CRLB values obtained from the hippocampal region of both groups. The independent *t*-test revealed significant differences in the cerebral metabolite concentrations between the two groups. The concentrations of GABA (2.064 ± 0.496 μmol/g vs. 2.538 ± 0.153 μmol/g; *p* = 0.019), Glu (6.693 ± 0.436 μmol/g vs. 7.387 ± 0.269 μmol/g; *p* = 0.001), and Glx (9.632 ± 0.323 μmol/g vs. 10.578 ± 0.632 μmol/g; *p* = 0.001) were significantly higher in CPR rats than in CTRL rats. The concentrations of mIns (4.138 ± 0.616 μmol/g vs. 3.132 ± 0.324 μmol/g; *p* = 0.001) and tCr (6.590 ± 0.298 μmol/g vs. 6.030 ± 0.478 μmol/g; *p* = 0.006) were significantly lower in CPR rats than in CTRL rats.

**Figure 4:**
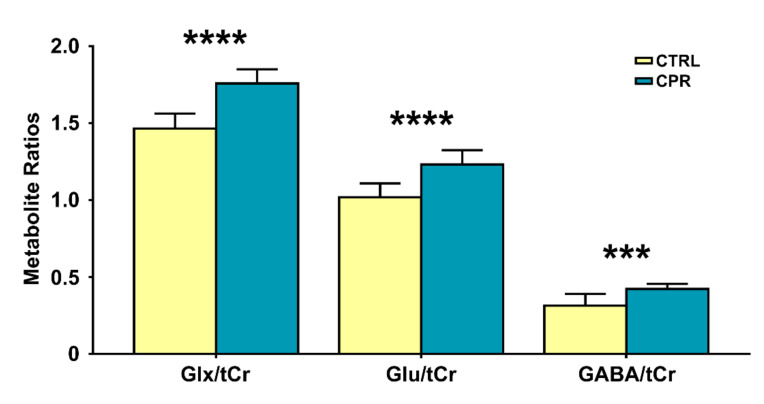
Bar graph showing the mean cerebral metabolite ratios in the right hippocampal region of the control (CTRL) and cuprizone-treated (CPR) rats. The vertical lines on each of the bars indicate the (+) standard deviation of the mean values. *** *p* < 0.005; **** *p* < 0.001. GABA, gamma-aminobutyric acid; Glx, Glu (glutamate) + Gln (glutamine); tCr (total Cr), Cr (creatine) + PCr (phosphocreatine).

Figure 4 illustrates the selected metabolite ratios of interest obtained from the hippocampal region of both groups. The Glx/tCr (1.465 ± 0.097 vs. 1.758 ± 0.091; *p* < 0.001), Glu/tCr (1.018 ± 0.090 vs. 1.231 ± 0.094; *p* < 0.001), and GABA/tCr (0.314 ± 0.076 vs. 0.422 ± 0.033; *p* = 0.002) ratios in CPR rats were significantly higher than those in CTRL rats.

## 4. Discussion

To date, numerous studies have investigated the extensive hippocampal demyelination seen in MS patients and animal models, in parallel with inflammation, microglia, and macrophage activation, which are strongly related to cognitive defects [7,23,24]. Notably, cerebral demyelination in the hippocampal region is considered a key feature in MS [7]. However, the underlying mechanisms of cuprizone-induced oligodendrocyte death and hippocampal damage caused by demyelination have not been well-revealed [9,11]. For these reasons, the present study demonstrates how axonal demyelination affects cerebral metabolites in the GM of the hippocampal region, quantified using high-resolution proton MR spectra.

In the present study, the concentrations of GABA, Glu, mIns, tCr, and Glx in the hippocampus of CPR rats were significantly altered compared to those in the hippocampus of CTRL rats. In addition, the Glx/tCr, Glu/tCr, and GABA/tCr ratios were statistically higher in CPR rats than in CTRL rats. These findings suggest that neurochemical abnormalities are present in the hippocampal region of cuprizone-treated rats.

Previous clinical ^1^H MRS studies have consistently shown a significant decrease in NAA concentrations and NAA/Cr ratios in the lesions of patients with MS [25,26]. The significant decrease in NAA concentrations is possibly representative of axonal damage and neuronal degeneration [27]. However, the present study showed that NAA concentrations were not significantly different between CPR and CTRL rats.

In our study, compared to CTRL rats, CPR rats exhibited significantly higher Glu concentrations and Glu/tCr ratios in the hippocampal region. In addition, the statistically significant difference in Glx concentrations and Glx/tCr ratios might reflect the significant difference in Glu concentrations. Previous studies have indicated that the increased concentrations of Glu in acute MS lesions may be related to glutamate excitotoxicity induced by neuronal inflammation [13,27]. The oligodendrocytes in myelinating axons are especially vulnerable to increased tissue glutamate concentration and excitotoxicity [23]. The death of oligodendrocytes in the CNS is accompanied by microglial and macrophage activation, astrogliosis, and inflammatory cytokine release [28,29,30]. In particular, large quantities of Glu are produced and released by activated macrophages and microglial cells [27,31]. Therefore, increased Glu concentration in the demyelinated hippocampus may lead to Glu excitotoxicity, possibly due to neuronal inflammation and microglial and macrophage activation.

GABA is a principal neurotransmitter and is naturally present in relatively lower concentrations in the brain than other metabolites [32]. In particular, the non-invasive measurements of GABA concentration have limited reliability due to severely overlapping peaks (NAA, Glu, Gln, and Cr) in the narrow chemical shift, and it is difficult to isolate it at a main magnetic field below 3 T in strength [33]. For these reasons, studies on the detection of cerebral GABA concentrations in vivo using ^1^H MRS in patients with MS and animal models are sparse [13,34,35]. To date, only a few clinical studies in patients with MS have shown significantly altered GABA concentrations in the hippocampus [36,37], posterior cingulate cortex [33,36], parietal lobe [38], and sensorimotor cortex [38,39] using the Mescher–Garwood (MEGA)-PRESS editing technique.

The present study found significantly higher GABA concentrations and GABA/tCr ratios in the hippocampus of CPR rats than in the hippocampus of CTRL rats. According to previous in vitro studies, GABA and related receptors mainly exist in neurons, glial cells, and immune cells [40,41]. Numerous studies have demonstrated the immunosuppressant and anti-inflammatory properties of elevated GABA concentrations in patients with MS and animal models of neuro-inflammatory damage [38,40,42,43]. Thus, higher GABA concentrations and ratio values may reflect the corrective response to alleviate neuronal inflammation in the hippocampal region following cuprizone-induced demyelination.

Adenosine triphosphate (ATP) is the main source of energy for cell metabolism and is involved in cellular processes in the brain parenchyma [44]. In the presence of ATP, which is generated by oxidative phosphorylation in the mitochondria, creatine kinase (CK) catalyzes the reversible transphosphorylation between ATP and Cr to ADP and PCr [45]. These reactions can help balance the levels of phosphorylated and non-phosphorylated substrates and stabilize the energy supply system [46]. Previous studies have demonstrated that the CK isoform and Cr can protect mitochondrial permeability transition pores and, thus, have an important anti-apoptotic effect [47]. Moreover, CK and Cr help inhibit the generation of reactive oxygen species (ROS) within the mitochondria by facilitating the recycling of adenosine diphosphate during periods of increased glucose utilization [48]. In the present study, CPR rats exhibited significantly lower tCr concentrations in the hippocampal region than those in CTRL rats. Although there were no significant differences in Cr and PCr concentrations between the two groups, a trend for lower concentrations in CPR rats was observed. A previous study suggested that decreased tCr concentrations may reflect an energy deficit in brain neurons, possibly due to mitochondrial dysfunction [49]. Therefore, the significantly lower tCr concentrations found in this study might be due to mitochondrial dysfunction caused by cuprizone-induced demyelination in the hippocampus.

Our key finding was that mIns concentrations in the hippocampal region were significantly lower in CPR rats than in CTRL rats. Previous studies have suggested that mIns originates from intracellular astrocyte stores and is considered a key marker of astrocytic activation and proliferation [50,51,52]. Moreover, decreased mIns concentrations might reflect astrocytic necrosis with hypo-osmolarity [53] and oligodendrocyte injury [51]. Fernando et al. reported that significantly higher mIns concentrations were observed in normal-appearing white matter (NAWM) of clinically isolated syndrome patients than in NAWM of healthy subjects [54]. They suggested that the higher mIns concentrations may be due to the extensively increased gliosis in demyelinated lesions and NAWM [54]. Llufriu et al. suggested that decreased NAA concentrations and increased mIns concentrations indicate reduced axonal integrity and increased gliosis, respectively. Therefore, the mIns/tNAA ratio can be useful for predicting brain atrophy and sustained clinical progression [50,52,54]. Although the present study found no significant difference in tNAA concentrations between the two groups, alterations in mIns concentrations may reflect astrocytic necrosis and astrogliosis, which have cardinal importance in the progression of MS [52].

Our study has some limitations. The present study examined only one region of the hippocampus and thus cannot suggest multi-regional changes and distributions in the neurochemical profile. To address these limitations, further studies should investigate how to visualize and quantify the distribution of metabolite changes based on the molecular process in vivo. In recent years, numerous studies have been proposed for detecting neurochemical signals such as Glu [55], mIns [56], Cr [57], and GABA [19] compounds, using the chemical exchange saturation transfer (CEST) technique. CEST imaging is a novel contrast enhancement technique that enables the indirect measurement of molecules with exchangeable solute protons [58]. Thus, CEST imaging with specific metabolites will be suitable for better understanding the molecular mechanism of cerebral demyelination in our future studies.

In our early study, we did not examine long-term changes in brain metabolites in an animal model of cuprizone-induced demyelination and remyelination. Therefore, we cannot provide neurochemical information on the effects of long-term demyelination or the evaluation of the remyelination process. Hence, additional studies are needed to examine the changes in metabolites related to therapeutics efficacy that can accelerate remyelination and neuronal recovery.

## 5. Conclusions

In summary, to explore the neurochemical profile of cuprizone-induced demyelination in the hippocampus, we conducted an in vivo ^1^H MRS study at 7 T. The present study highlights GABA, Glu, Glx, tCr, and mIns concentrations as in vivo measures that are of potential relevance in studying hippocampal demyelination. Therefore, several significantly altered metabolites are possibly utilized as a neuronal marker in cuprizone-induced demyelination, and these findings might have cardinal importance in the progression of MS.

## Data Availability

The data that support the findings of this study are available from the corresponding author upon reasonable request.
